# Live attenuated influenza vaccine effectiveness against hospitalisation due to laboratory-confirmed influenza in children two to six years of age in England in the 2015/16 season

**DOI:** 10.2807/1560-7917.ES.2017.22.4.30450

**Published:** 2017-01-26

**Authors:** Richard Pebody, Bersabeh Sile, Fiona Warburton, Mary Sinnathamby, Camille Tsang, Hongxin Zhao, Joanna Ellis, Nick Andrews

**Affiliations:** 1 National Infection Service, Public Health England, London, United Kingdom

**Keywords:** influenza, vaccine effectiveness, hospitalisation, LAIV

## Abstract

The United Kingdom is introducing a universal annual influenza vaccination programme for children. Live attenuated influenza vaccine (LAIV) effectiveness (VE) against laboratory-confirmed influenza hospitalisation in 2 to 6 year-olds in England was measured in 2015/16 using the screening method. VE adjusted for age, geography and month was 54.5% (95% confidence interval (CI): 31.5% to 68.4%) for all influenza types combined; 48.3% (95% CI: 16.9% to 67.8%) for A(H1N1)pdm09 and 70.6% (95% CI: 33.2% to 87.1%) for B. The findings support on-going programme roll-out.

## Introduction

The United Kingdom (UK) started the phased introduction of a universal paediatric influenza vaccination programme in 2013/14, following recommendations from the UK Joint Committee on Vaccination and Immunisation (JCVI) [1]. The programme will ultimately be targeted at all children 2 to 16 years of age, with the offer of a single dose of a newly licensed live attenuated influenza vaccine (LAIV) to healthy children. The programme aims to both directly protect the children themselves, but also by reducing their ability to spread influenza, protect other vulnerable members of the population. The programme initially targeted all 2 and 3 year-olds across the UK with trivalent LAIV, and by 2015/16, had extended to all children aged 2 to 4 years of age plus school years 1 and 2 (5 and 6 years of age) in England with quadrivalent LAIV [2].

The UK has published a series of papers demonstrating that the programme has provided direct protection against influenza-confirmed infection in primary care over the first three seasons [3,4]. The UK has published evidence that LAIV provided significant protection against influenza for children consulting in primary care in 2015/16 [4], however, to date no data have been published on the potential effectiveness of this vaccine against more severe disease. The UK Severe Influenza Surveillance System (USISS) was established after the 2009 influenza pandemic and collects information on laboratory-confirmed influenza hospitalisations through a sentinel network of acute hospital trusts in England [5]. This surveillance system provides an opportunity to measure whether the new paediatric influenza vaccination programme also provides direct protection against more severe infection in children.

## Methods

We used the screening method to estimate vaccine effectiveness (VE) in vaccine-eligible children aged 2 to 6 years in England in the 2015/16 season, comparing vaccination coverage in children hospitalised with laboratory-confirmed influenza infection to vaccination coverage in children in the general population. This approach has been described elsewhere [6,7].

A case was defined as a child aged 2 to 6 years on 1 September 2015, and thus eligible for influenza vaccination, and reported to be hospitalised with laboratory-confirmed influenza infection by reverse transcription real-time PCR (RT-PCR) in the period between week 40 2015 and week 20 2016.

Cases were identified from the USISS, a national surveillance system which collects individual level reports on laboratory-confirmed hospitalisations of influenza in children from a sentinel laboratory network in England [5]. Cases’ general practitioners (GP) were sent a postal questionnaire to identify whether the cases had received influenza vaccination during the 2015/16 campaign and if so, the vaccination date and whether the vaccine was administered by injection or intranasally. Finally phone contact was made with non-responding practices.

A child was classified as vaccinated if they received at least one dose of influenza vaccine at least 14 days before the child’s date of reported symptom onset, as this was considered the minimum time period for the child to achieve maximum protection. If the child was vaccinated less than 14 days before onset or had an unknown vaccination record then the child was not considered in the analysis. Cases vaccinated by injection (i.e. by injected inactivated vaccine; IIV) were also excluded. This information was used to determine the proportion of cases vaccinated (PCV).

Seasonal influenza vaccination coverage (PPV) for the population of children 2 to 6 years of age on 1 September 2015 in England was identified through a national electronic reporting system (Immform). This is a web-based system developed to collect data on influenza vaccine uptake in near real time during the influenza season. Data are collected from all GP practices on a monthly basis online using almost entirely fully automated data extraction methods. These include seasonal influenza vaccination for children 2 to 4 years of age [8]. Data were extracted from Immform each month on the number of children registered in primary care, and number of children who received seasonal influenza vaccination between 1 September 2015 and 31 January 2016. Immform does not distinguish whether LAIV or IIV was administered (a small number of children will have received IIV if they are contraindicated because of severe asthma or egg allergy or immunosuppression). Immform data were extracted at the end of each month from GP information systems and were available by year of age. 

In addition, cumulative monthly uptake in children of school year 1 and 2 (5 and 6 years of age) was available across England through a separate manual reporting system into Immform. Local teams undertaking school-based campaigns report the number of eligible registered children and number of children who received influenza vaccine to Immform. Monthly data were also available for this collection for the period between 1 September 2015 and 31 January 2016 [9].

Coverage data for all age groups were available each month at the Local Authority (LA) and Regional level.

Cases included in the analysis were described by age at September 2015 (2–4 and 5–6 years), month of infection (September 2015 to April 2016), LA of residence (unless this information was missing, in which case the LA of their GP practice was used), influenza A(H3N2), A(H1N1)pdm09 and B and influenza vaccine status (intranasal vaccine, unvaccinated) in 2015/16. Information was not available on risk group status for cases.

Crude VE for hospitalised influenza cases were estimated as: 

1- PCV_all_/(1-PCV_all_)

     PPV_all_/(1-PPV_all_)

Where PCV_all_ is the overall proportion of cases vaccinated and PPV_all_ the overall end of season population coverage.

Adjusted VE for hospitalised influenza cases was estimated by obtaining the PPV that matched to each case according to LA, age at 1 September 2015 and the end of the month closest to two weeks before hospital admission (the 2 weeks is to allow time for protection following vaccination). This was undertaken for all circulating influenza: influenza A(H1N1)pdm09 and influenza B (the dominant circulating strains). Adjusted VE was then estimated from a logistic regression model where the matched log of (PPV/(1-PPV)) was used as an offset and the outcome was vaccination status for each case, an approach described previously [6,7].

This work was undertaken as a routine public health function to monitor vaccination programmes; Public Health England (PHE) holds permissions to collect data under Section 251 of the National Health Service Act 2006 and the 2002 Health Service (Control of Patient Information) regulations as part of monitoring the performance of the national vaccination programme.

## Results

There were a total of 176 children 2 to 6 years of age on 1 September 2015 with laboratory-confirmed influenza infection reported to USISS, who were hospitalised between week 40 2015 and week 20 2016. Response was received from GPs for all the cases. Nineteen cases were excluded (11%), five due to unknown vaccination status in the returned questionnaire; one with no hospital admission date; one that was vaccinated within 14 days of admission; 11 that had received IIV and one that had vaccine type unknown. This left 157 cases for analysis. There were 10 cases where vaccination date was unknown but they were assumed vaccinated at more than 14 days before onset as all were hospitalised after mid-January, when the vast majority of vaccinations had been completed.

Of these 157 included cases, overall 99 (63.1%) tested positive for influenza A(H1N1)pdm09, 14 (8.9%) for influenza A (subtype unspecified) and 44 (28.0%) for influenza B. Median age at time of influenza infection was 4 years.

Overall 34 cases (21.7%) had received LAIV in 2015/16; the median interval between vaccination and date of onset of illness for those with information available was 120 days (range: 16–173 days).

Nationally in 2015/16, 1,367,957 of 3,431,319 (39.9%) children 2 to 6 years had received seasonal influenza vaccination. Coverage is shown in Table 1 by age group and month.

**Table 1:** Cumulative live attenuated influenza vaccine^a^ uptake by age group and month, England, 1 September 2015–31 January 2016 (n=3,431,319 persons)

Age group in years	Per cent (N children vaccinated/N children registered)			
	1 Sep 2015–31 Oct 2015	1 Sep 2015–30 Nov 2015	1 Sep 2015–31 Dec 2015	1 Sep 2015–31 Jan 2016
**2–4**	16.7% (320,013/1,920,171)	26.9% (550,382/2,048,535)	30.9% (642,106/2,077,665)	31.5% (661,423/2,098,909)
**5–6**	11.7% (148,480/1,264,339)	40.3% (530,610/1,317,526)	50.2% (668,072/1,331,241)	53.0% (706,534/1,332,410)
**Total** **2–6**	14.7% (468,493/3,184,510)	32.1% (1,080,992/3,366,061)	38.4 (1,310,178/3,408,906)	39.9% (1,367,957/3,431,319)

^a^ The paediatric influenza vaccination programme in England offers a single dose of live attenuated influenza vaccine (LAIV) to all healthy children. A small number of children will have received inactivated influenza vaccine (IIV) if they are contraindicated because of severe asthma or egg allergy or immunosuppression, Information on IIV uptake is unavailable.

The crude and adjusted VE for preventing influenza hospitalised cases in healthy children by age group and by influenza type is shown in Table 2. Crude overall VE was 58.3% for all influenza types, which decreased to 54.5% after adjusting for geography, month and age. Results after stratifying by influenza A(H1N1)pdm09 and B gave an adjusted VE in children 2 to 6 years of age of 48.3% for influenza A(H1N1)pdm09 and 70.6% for influenza B. There was no significant difference on stratifying by age group.

**Table 2:** Crude and adjusted live attenuated influenza vaccine effectiveness by age group, England, 1 September 2015–22 May 2016

Age group in years	Influenza type	PCV	Crude VE (95%CI)	Adjusted VE^a^ (95%CI)
**2–4**	Any influenza	29/133 (21.8%)	39.4% (7.7% to 61.3%)	49.6% (23.6% to 66.7%)
	(H1N1)pdm09	19/84 (22.6%)	36.5% (-7.6% to 64.0%)	46.7% (10.7% to 68.2%)
	B	6/36 (16.7%)	56.4% (-6.1% to 85.1%)	66.0% (17.9% to 85.9%)
**5–6**	Any influenza	5/24 (20.8%)	76.7% (35.3% to 93.2%)	69.6% (15.9% to 86.4%)
	(H1N1)pdm09	4/15 (26.7%)	67.7% (-8.8% to 92.5%)	55.6% (-45.2% to 86.4%)
	B	1/8 (12.5%)	87.3% (1.2% to 99.7%)	84.9% (-30.1% to 98.3%)
**Total** **2–6**	Any influenza	34/157 (21.7%)	58.3% (38.8% to 72.4%)	54.5% (31.5% to 68.4%)
	(H1N1)pdm09	23/99 (23.2%)	54.5% (26.5% to 72.8%)	48.3% (16.9% to 67.8%)
	B	7/44 (15.9%)	71.5% (35.1% to 89.4%)	70.6% (33.2 to 87.1%)

CI: confidence interval; PVC: proportion of cases vaccinated; VE: vaccine effectiveness.

^a^ Adjusted VE by local authority, month of infection and age in years.

## Discussion

Our study finds evidence that quadrivalent LAIV administered to children 2 to 6 years of age in England in 2015/16 was effective in preventing laboratory-confirmed influenza hospitalisation. We demonstrate good overall protection, including against both A(H1N1)pdm09 and influenza B.

There are a number of potential strengths and weaknesses to our study. The screening method is a well-recognised observational study design which has the potential to provide rapid and economical estimates of VE. However, it is recognised to have a number of potential limitations: firstly, VE estimates can be biased, if the cases arise from a population that differs from the population used to determine coverage rates; secondly if important confounders remain unadjusted and finally if vaccine information is incomplete. In this study, we have attempted to minimise the potential bias highlighted in the first point by comparing the vaccine coverage among cases to the uptake in the general population of the same age in the local area where cases lived. For the second point, we have adjusted for the main confounders identified in other influenza VE studies, namely age, time of infection and place of residence. Although we did not have information on risk-groups status for the cases and were not able to adjust for this potential confounder, we have not found it to be an important confounder previously for studies in primary care [3]. It is also important to note that influenza vaccine was offered to all children in these age groups. Nevertheless if cases belonged to risk groups and coverage was higher in those in risk groups, then we may have underestimated effectiveness. We assessed this further by increasing matched coverage by 5% which in turn increased VE estimates by ca 8%. For the final point on vaccine status, information on vaccination status of cases was ascertained from the patients’ records by their GPs and was almost complete. Although population information on type of vaccine administered was not available, the proportion of vaccinated children in the general population who received IIV was small, as this would only be children with severe asthma, egg allergy or immunosuppression who were contraindicated LAIV. 

Our findings of quadrivalent LAIV effectiveness for protection against influenza-related hospitalisation in 2015/16 are consistent with recently published findings from the UK which found that LAIV also provided significant protection against laboratory-confirmed influenza in primary care in 2 to 17 year-olds, with a similar effectiveness of 57.6% (95% confidence interval (CI): 25.1% to 76.0%) [4]. It is also encouraging that our findings of protection against severe disease with the screening method are congruent with results of a study reported from Scotland also for the 2015/16 season, but which rather used linked hospitalisation data and a cohort design [10]. In addition, the results are also consistent with those from Finland, where LAIV was also offered to children in 2015/16 and evidence of effectiveness was found in preventing laboratory-confirmed infection [11]. On the other hand, our results of significant protection are at odds with those reported from North America, where recent studies have suggested no evidence of significant effectiveness of LAIV in children against medically attended laboratory-confirmed influenza over the same period [12].

The VE findings for influenza A(H1N1)pdm09 demonstrate significant protection against influenza A(H1N1)pdm09 confirmed hospitalisation. Again these results are congruent with the UK study undertaken in primary care using the test-negative case–control approach [4]. The LAIV VE finding for influenza A(H1N1)pdm09 in this paper of 48.3% (95% CI: 16.9% to 67.8%) is consistent with previous hospital based studies. A study undertaken using the test-negative design in 2013/14 found an effectiveness of influenza vaccine against A(H1N1)pdm09 confirmed hospitalisation of 42.8% (95% CI: 6.3% to 65.0%) [13]. The findings presented here are encouraging particularly in light of the very poor vaccine effectiveness of LAIV against influenza A(H1N1)pdm09 noted recently in the US. The reasons for this apparent discordance between the UK, and other countries such as Finland using LAIV, and the US remain under investigation. It could relate to the vaccine itself, the circulating viruses or the population and their prior exposure [14,15]. The 2015/16 season in the UK, as in North America, was dominated by circulation of influenza A(H1N1)pdm09 strain, which was antigenically well matched to the A/Bolivia/559/2013 (A/California/7/2009-like) vaccine strain. Others authors have suggested that the US results and the relative reduction in A(H1N1)pdm09 effectiveness for LAIV compared with IIV in a range of settings, including the UK, is related to reduced replicative fitness of the A(H1N1)pdm09 LAIV A/Bolivia/559/2013 strain [15], although that factor alone would not explain the discordance of the US CDC with the UK and other results in both primary and secondary care. This may relate to country specific issues such as prior vaccination, or how the vaccine is handled. Further work is required to understand, what role each of these factors might be playing in contributing to the current observations. Nonetheless, the vaccine manufacturer has acknowledged these findings and is working to identify a more effective A(H1N1)pdm09 LAIV strain for potential incorporation to the 2017/18 LAIV vaccine [15].

Finally the LAIV VE influenza B finding in this paper is also consistent with the 2015/16 UK study of LAIV in primary care [4]. Although the UK experienced influenza B circulation mainly of the B/Victoria lineage, there were also some circulating viruses of the B/Yamagata-lineage [16]. As LAIV in 2015/16 was a quadrivalent vaccine containing both a B/Phuket/3073/2013-like virus and B/Brisbane/60/2008-like virus, both well matched to the circulating strains, this presumably explains the relatively high VE.

In summary, we have demonstrated that in 2015/16, LAIV provided moderate protection against laboratory-confirmed influenza infection resulting in hospitalisation in England, including against A(H1N1)pdm09 infection and also influenza B. The findings support the recommendation of the JCVI for the on-going roll-out of the UK paediatric programme [17]. Close ongoing monitoring will be critical to provide assurance that these positive findings are maintained, particularly in the light of the recent US observations.

## References

[1] Hakin B, Cosford P, Harvey F. The flu immunisation programme 2013/14 – extension to children. London: Department of Health; 26 Jul 2013. Available from: https://www.gov.uk/government/uploads/system/uploads/attachment_data/file/225360/Children_s_flu_letter_2013.pdf

[2] Public Health England (PHE). Flu plan. Winter 2015/16. London: PHE; May 2016. Available from: https://www.gov.uk/government/uploads/system/uploads/attachment_data/file/526143/Flu_Plan_Winter_2015_to_2016superseded.pdf

[3] Pebody R Warburton F Andrews N Ellis J von Wissmann B Robertson C Effectiveness of seasonal influenza vaccine in preventing laboratory-confirmed influenza in primary care in the United Kingdom: 2014/15 end of season results. Euro Surveill. 2015;20(36):30013. 10.2807/1560-7917.ES.2015.20.36.30013 26535911

[4] Pebody R Warburton F Ellis J Andrews N Potts A Cottrell S Effectiveness of seasonal influenza vaccine for adults and children in preventing laboratory-confirmed influenza in primary care in the United Kingdom: 2015/16 end-of-season results. Euro Surveill. 2016;21(38):30348. 10.2807/1560-7917.ES.2016.21.38.30348 27684603 PMC5073201

[5] Bolotin S Pebody R White PJ McMenamin J Perera L Nguyen-Van-Tam JS UK Severe Influenza Surveillance System (USISS) Steering Group A new sentinel surveillance system for severe influenza in England shows a shift in age distribution of hospitalised cases in the post-pandemic period. PLoS One. 2012;7(1):e30279. 10.1371/journal.pone.0030279 22291929 PMC3264602

[6] Farrington CP Estimation of vaccine effectiveness using the screening method. Int J Epidemiol. 1993;22(4):742-6. 10.1093/ije/22.4.742 8225751

[7] Thomas HL Andrews N Green HK Boddington NL Zhao H Reynolds A Estimating vaccine effectiveness against severe influenza in England and Scotland 2011/2012: applying the screening method to data from intensive care surveillance systems. Epidemiol Infect. 2014;142(1):126-33. 23591102 10.1017/S0950268813000824 PMC9152609

[8] Public Health England (PHE). Influenza immunisation programme for England, GP patient groups, Season 2015 to 2016. London: PHE; May 2016. Available from: https://www.gov.uk/government/uploads/system/uploads/attachment_data/file/544552/Seasonal_flu_GP_patient_groups_annual_report_2015_2016.pdf

[9] Public Health England (PHE). National Childhood Influenza Vaccination Programme 2015 to 2016, Seasonal influenza vaccine uptake for children of primary school age. London: PHE; May 2016. Available from: https://www.gov.uk/government/uploads/system/uploads/attachment_data/file/544542/Childhood_Influenza_Vaccination_Programme_Report_2015_2016.pdf

[10] Health Protection Scotland (HPS). Flu vaccine effectiveness in Scottish primary school age children from the 2015/16 season. Glasgow: HPS. [Accessed 25 Jan 2017]. Available from: http://www.hps.scot.nhs.uk/resourcedocument.aspx?id=5529

[11] Nohynek H Baum U Syrjänen R Ikonen N Sundman J Jokinen J Effectiveness of the live attenuated and the inactivated influenza vaccine in two-year-olds - a nationwide cohort study Finland, influenza season 2015/16. Euro Surveill. 2016;21(38):30346. 10.2807/1560-7917.ES.2016.21.38.30346 27684447 PMC5073199

[12] Grohskopf LA Sokolow LZ Broder KR Olsen SJ Karron RA Jernigan DB Prevention and Control of Seasonal Influenza with Vaccines. MMWR Recomm Rep. 2016;65(5):1-54. 10.15585/mmwr.rr6505a1 27560619

[13] Rondy M Castilla J Launay O Costanzo S Ezpeleta C Galtier F Moderate influenza vaccine effectiveness against hospitalisation with A(H3N2) and A(H1N1) influenza in 2013-14: Results from the InNHOVE network. Hum Vaccin Immunother. 2016;12(5):1217-24. 10.1080/21645515.2015.1126013 27065000 PMC5036960

[14] Penttinen PM Friede MH Decreased effectiveness of the influenza A(H1N1)pdm09 strain in live attenuated influenza vaccines: an observational bias or a technical challenge? Euro Surveill. 2016;21(38):30350. 10.2807/1560-7917.ES.2016.21.38.30350 27684999 PMC5073203

[15] Ambrose CS Bright H Mallory R Letter to the editor: Potential causes of the decreased effectiveness of the influenza A(H1N1)pdm09 strain in live attenuated influenza vaccines. Euro Surveill. 2016;21(45):30394. 10.2807/1560-7917.ES.2016.21.45.30394 27918259 PMC5144940

[16] Public Health England (PHE). Surveillance of influenza and other respiratory viruses in the United Kingdom: Winter 2015 to 2016. London: PHE; May 2016. Available from: https://www.gov.uk/government/uploads/system/uploads/attachment_data/file/526405/Flu_Annual_Report_2015_2016.pdf

[17] Joint Committee on Vaccination and Immunisation (JCVI). JCVI minutes of the meeting, 23 August 2016. JCVI. [Accessed 25 Jan 2017]. Available from: https://app.box.com/s/iddfb4ppwkmtjusir2tc/1/2199012147/98304591342/1

